# Olfactory dysfunction: A plausible source of COVID-19-induced neuropsychiatric symptoms

**DOI:** 10.3389/fnins.2023.1156914

**Published:** 2023-03-20

**Authors:** Alan Pui-Lun Tai, Mei-Kei Leung, Benson Wui-Man Lau, Shirley Pui-Ching Ngai, Way Kwok-Wai Lau

**Affiliations:** ^1^Department of Special Education and Counselling, The Education University of Hong Kong, Hong Kong, Hong Kong SAR, China; ^2^Integrated Centre for Wellbeing, The Education University of Hong Kong, Hong Kong, Hong Kong SAR, China; ^3^Bioanalytical Laboratory for Educational Sciences, The Education University of Hong Kong, Hong Kong, Hong Kong SAR, China; ^4^Department of Counselling and Psychology, Hong Kong Shue Yan University, Hong Kong, Hong Kong SAR, China; ^5^Department of Rehabilitation Sciences, The Hong Kong Polytechnic University, Hong Kong, Hong Kong SAR, China

**Keywords:** COVID-19, neuropsychiatric symptoms, nervus terminalis, olfactory system, *trans*-neuronal viral transmission

## Abstract

Olfactory dysfunction and neuropsychiatric symptoms are commonly reported by patients of coronavirus disease 2019 (COVID-19), a respiratory infection caused by severe acute respiratory syndrome coronavirus 2 (SARS-CoV-2). Evidence from recent research suggests linkages between altered or loss of smell and neuropsychiatric symptoms after infection with the coronavirus. Systemic inflammation and ischemic injury are believed to be the major cause of COVID-19-related CNS manifestation. Yet, some evidence suggest a neurotropic property of SARS-CoV-2. This mini-review article summarizes the neural correlates of olfaction and discusses the potential of *trans*-neuronal transmission of SARS-CoV-2 or its particles within the olfactory connections in the brain. The impact of the dysfunction in the olfactory network on the neuropsychiatric symptoms associated with COVID-19 will also be discussed.

## 1. Introduction

Coronavirus Disease 2019 (COVID-19) is caused by Severe acute respiratory syndrome coronavirus-2 (SARS-CoV-2). Since its outbreak in 2019, over 650 million confirmed cases were reported globally (World Health Organization, 2023). The commonly reported symptoms included cough, fever, fatigue, loss of taste or smell, sore throat, headache, aches, diarrhea, rash on skin, red or irritated eyes, and shortness of breath (World Health Organization, 2023). Emerging evidence reports the impact of COVID-19 on influencing taste and smell, not only in the acute phase but also extending to the recovery phase. Previous literature hypothesized that such dysfunction could be related to the influence of SARS-CoV-2 *via* its binding to angiotensin-converting enzyme-2 (ACE2) receptors, an entry protein for SARS-CoV-2 (Zhou et al., 2020), on mucous membranes, primarily in the olfactory epithelia (Bourgonje et al., 2020). The surface expression of ACE2 protein was reported to be more remarkable in lung alveolar epithelial cells and enterocytes of small intestine (Hamming et al., 2004). The inhaled virus binds to the ACE2 receptors in epithelial cells in the nasal cavity and further propagates to the respiratory tract (Mason, 2020). The expression of SARS-CoV-2 entry protein ACE2 in airway epithelial cells was found to be increased 3 times in patients with COVID-19 (Chua et al., 2020). Infected epithelial cells secrete chemokines that trigger the migration of different immune cell populations including neutrophils, T cells and mast cells to the site and cause further damage to the epithelium. Analysis of RNA-seq further demonstrated that type II alveolar cells, myocardial cells, proximal tubule cells of kidney, ileum and esophagus epithelial cells and urothelial cells of bladder were vulnerable to the manifestation of different organ infections or damage (Zou et al., 2020) which supports the potential impacts of SARS-CoV-2. Torabi et al. (2020) reported that COVID-19 patients had significantly elevated Tumor necrosis factor α (TNF-α) levels in the olfactory epithelium, which may induce direct inflammation and contribute to the acute olfactory loss described in many COVID-19 patients. Other than invading through ACE2 receptors, it is hypothesized that viral invasion could access the central nervous system (CNS) through a hematogenous route [blood brain barrier (BBB)], neuronal retrograde dissemination route (peripheral neurons) or transcribial routes [olfactory bulb or cerebral spinal fluid (CSF) (Baig and Sanders, 2020]. Being determined by the infection of SARS-CoV-2, COVID-19 is a multi-systemic disease. Different systems like respiratory, cardiovascular, nervous, renal, and digestive systems are involved in the disease. Multiple signs and symptoms, including widespread inflammatory response, cytokine storm, and abnormalities of blood cells, are possible in COVID-19. Due to the hyperinflammation and other abnormalities, the nervous system may be affected (Temgoua et al., 2020). In this review, details of how COVID-19 influences olfactory function and its associated mechanistic pathways in damaging the CNS will be discussed.

## 2. Olfaction and its neural correlates

Olfaction is one of the critical senses for humans to interact with the world. In the olfactory system, olfactory stimuli are received by first-order sensory neurons embedded in the olfactory epithelium located in the upper side of the nasal cavity, which then pass information to the olfactory bulb in the brain at the base of the frontal lobe (Firestein, 2001; Shipley et al., 2003; Doty, 2009). The olfactory bulb is a vital intermediate relay station of the olfactory pathway, which passes the olfactory information to the brain. The olfactory bulb projects information to the primary olfactory cortex *via* the olfactory tract, which is formed by the fibers from the output neurons, namely mitral and tufted cell axons (Shipley et al., 2003; Doty, 2009; Zhou et al., 2019). The primary olfactory area consists of numerous cortical and subcortical regions of the brain, including the anterior olfactory nucleus, the piriform cortex (posterior orbitofrontal cortex; stocktickerOFC), parts of the amygdala, the olfactory tubercle, the frontal and temporal piriform cortices, etc. (Doty, 2009; Zhou et al., 2019; Cersosimo et al., 2021). The primary olfactory cortex directly projects information to the secondary olfactory regions, which include the thalamus, hypothalamus, hippocampus, and stocktickerOFC (Doty, 2009; Zhou et al., 2019; Cersosimo et al., 2021).

## 3. Olfactory dysfunction

Olfactory dysfunction can be a total loss of smell (anosmia), an incomplete loss of smell (partial anosmia, hyposmia, or microsmia), and distortion of smell (dysosmia), a presence of a scent without stimulus (phantosmias); the inability to recognize odors (olfactory agnosia) (Doty, 2009; Han et al., 2019). OD can be bilateral or unilateral (Doty, 2009); thus, some individuals with unilateral OD may not be aware and diagnosed immediately. Around 29% of the population suffers from OD (Desiato et al., 2021), in which older men have the highest prevalence (>55 years; 34.5%) (Desiato et al., 2021). Besides age, upper respiratory infections, brain trauma, and sinonasal disease can also cause olfactory loss (Temmel et al., 2002; Doty, 2009). Neurological disorders are also a common cause of OD (Doty, 2009; Han et al., 2019). Most recently, COVID-19 is also found to be related to OD (Whitcroft and Hummel, 2020).

Olfactory dysfunction is usually associated with structural and functional changes of the brain. The volume of the olfactory bulb is positively correlated with the olfactory function, supported by a large body of research (Yousem et al., 1998; Rombaux et al., 2006; Buschhüter et al., 2008; Seubert et al., 2013; Mazal et al., 2016; Han et al., 2018). A decrease in the gray matter volume was also reported across the primary and the secondary olfactory cortex in individuals suffering from OD compared to healthy controls (Han et al., 2018, 2019). One of the explanations for these structural changes is the decreased sensory input due to olfactory loss (Bitter et al., 2010). Reduction in white matter connectivity in olfactory brain regions (Ibarretxe-Bilbao et al., 2010; Erb et al., 2012; Erb-Eigner et al., 2014) and between the corpus callosum and the superior longitudinal fasciculi (Segura et al., 2013) was also associated with OD. A reduction of fiber connections in different areas of the brain could be reflecting a common cause of degeneration, like aging or other degenerative illness, which are highly related to OD (Segura et al., 2013).

The functional changes of the brain under OD can be classified into three types (Han et al., 2019). First, a widespread decrease in activation among olfactory-related brain regions, including the piriform cortex, amygdala, OFC, insula, and anterior cingulate cortex (Levy et al., 1998, 1999; Pellegrino et al., 2016; Han et al., 2018; Moon et al., 2018) was found in OD patients. These regions are responsible for encoding information (e.g., smell), cognitive-emotional processing, decision-making, and attention allocation. Second, top-down cognitive modulation moderates olfactory perception by higher levels of cognitive processing (Rolls, 2011), such as olfactory imagery, odor expectation, and odor-related words. Individuals with OD were found to be allocating more resources for odor imagery, resulting in a higher activation level in the dorsal lateral prefrontal cortex, cerebellum, and precuneus (Flohr et al., 2014). Similarly, higher activation was found in the left inferior frontal gyrus, insula, and bilateral angular gyrus for individuals with OD while expecting odor-related words (Han et al., 2020). Lastly, the change in the functional network is also found to be related to OD. Functional connectivity measured the temporal correlation of neuronal activity between different brain regions (Damoiseaux et al., 2006). A widespread reduction in functional connectivity in olfactory and non-olfactory networks has been found among individuals with OD (Murphy et al., 2005; Nigri et al., 2013; Kollndorfer et al., 2015; Su et al., 2015; Yoneyama et al., 2018). For example, reduced connectivity in the somatosensory and integrative networks was found in people with OD (Kollndorfer et al., 2015), which affects the performance and coordination of motor tasks as well as sensory integration processes. Connectivity between the regions in the olfactory brain areas like the anterior cingulate cortex, the entorhinal cortex, and the cerebellum were also found to be reduced among patients with OD (Kollndorfer et al., 2015; Su et al., 2015).

Being connected to multiple cortical and subcortical regions of the brain, the dysfunction of the olfactory system is related to several mental health problems, including schizophrenia, depression, and an early clinical sign of Alzheimer’s disease and Parkinson’s disease (Moberg et al., 1999; Doty, 2009; Yuan and Slotnick, 2014). With a 29% prevalence rate of OD (Desiato et al., 2021), its impact on neuropsychiatric diseases cannot be ignored. To better diagnose and rehabilitate patients with OD, the cause of it should be clearly identified. One of the common causes of OD discovered recently is due to the infection with SARS-CoV-2.

## 4. COVID-19-induced OD and neuropsychiatric symptoms

With the recent COVID-19 outbreak, the number of patients with OD increased. Based on a meta-analysis published in 2020, the prevalence rate for OD in COVID-19 patients was 43% (von Bartheld et al., 2020), which dropped globally to about one-tenth with the more recent Omicron variants of COVID-19 (von Bartheld and Wang, 2023). Some COVID-19 patients have long-lasting OD (Moein et al., 2020; Boscolo-Rizzo et al., 2022; Tan et al., 2022). Infection with SARS-CoV-2 could affect the olfactory bulb and other olfaction-related brain regions. The average volume of the olfactory bulb and tract was significantly reduced in COVID-19 patients compared with the control (Altunisik et al., 2021; Yildirim et al., 2022). Douaud et al. (2022) found that the gray matter thickness of the OFC and the parahippocampal gyrus decreased among COVID-19 cases, which echoes the histological findings that ischemic injury was observed through the hippocampal CA1 region and the surrounding parahippocampal region (Fabbri et al., 2021).

Coronavirus disease 2019 patients suffering from OD are more likely to develop psychological disabilities, when compared with patients without OD. In an online survey, among 322 COVID-19 cases experiencing OD, 43% also experienced depression (Coelho et al., 2021). Another study reported that COVID-19 patients who experienced OD had 30% more risk for suicidal thoughts and depression compared with those without OD (Yom-Tov et al., 2021). Higher anxiety scores were also reported from COVID-19 patients who experienced OD (Dudine et al., 2021). These findings indicate the association among COVID-19, OD and neuropsychiatric symptoms. The plausible ways of how SARS-CoV-2 could damage the CNS and result in neuropsychiatric manifestations will be discussed below.

## 5. CNS consequences of COVID-19

Growing evidence supports that SARS-CoV-2 can damage the CNS. Neuroinflammation, activation of microglia and neuronal death were found in postmortem cortex tissues of COVID-19 patients, and hyperemia of the meninges was observed in 90% of patients in an autopsy study (Boroujeni et al., 2021; Colombo et al., 2021). Mild neuropathological changes in formalin-fixed postmortem samples of COVID-19 patients, and pronounced neuroinflammatory changes in the brainstem suggested that the CNS damage was not directly caused by SARS-CoV-2 (Matschke et al., 2020). In animal studies using Syrian hamsters and non-human primates as models, the detection of SARS-CoV-2 viral particles in the olfactory pathway was associated with robust neuroinflammation and neuronal damage (Beckman et al., 2022; Käufer et al., 2022). Neuroinflammation could be sustained for a long period of time even after the acute phase of the disease. In fact, long-term deficits in olfactory function and neuropsychiatric deficits are observed in a significant proportion of individuals who recovered from COVID-19 (Badenoch et al., 2021; Doty, 2022). Such manifestations of multi-system symptoms after recovery from COVID-19 are termed “long-COVID” (Stefanou et al., 2022). More than one-third of patients reported long-COVID symptoms related to the nervous system (Stefanou et al., 2022), which includes fatigue, “brain fog,” cognitive dysfunction, alteration in gustation/olfaction and psychiatric manifestation like mood disturbances (Premraj et al., 2022; Stefanou et al., 2022). Persistent systemic inflammation and the presence of viral RNA in the brain of COVID-19 patients after a prolonged period are considered a plausible cause of long-COVID manifestation of neuropsychiatric symptoms (Stein et al., 2022). Nevertheless, there are different hypotheses suggesting the routes of entry to the CNS. For instance, SARS-CoV-2 was suggested to enter the brain through invasion of enterocytes of the gut where direct connection of the enteric nervous system with the brain are made *via* the vagus nerve (Gao et al., 2020). Nagu et al. (2020) proposed another route that is commonly adopted by other viruses including coronavirus, which is by infecting the leukocytes for transporting the virus across the BBB, triggering the release of proinflammatory cytokines and chemokines that increases the permeability of BBB, hence facilitating the entry of SARS-CoV-2 to the CNS and causing damage. The olfactory bulb and neurons were proposed to be an important site for SARS-CoV-2-induced CNS damage (Wu et al., 2020). All these paths involve the binding of the spike protein from the coronavirus to the ACE2 receptor on the target cells, which is abundantly expressed on various cell types including nerve cells (Iroegbu et al., 2020). Due to the vicinity of the olfactory bulb and neurons with the brain, it was believed that the olfactory bulb could be the first site of neuroinvasion by SARS-CoV-2. However, existing evidence has questioned this claim.

## 6. Routes of viral invasion

Although the olfactory bulb has direct neural connection to the olfactory sensory epithelium in the nasal cavity (Meinhardt et al., 2021; Xydakis et al., 2021; Lopez et al., 2022), neuroinvasion associated with SARS-CoV-2 is less likely to be initiated through infection at the olfactory bulb. In the olfactory mucosa, ACE2 and neuropilin-1 are highly expressed, providing cellular access points for SARS-CoV-2 (Butowt and Bilinska, 2020; Cantuti-Castelvetri et al., 2020). In contrast, ACE2 is not expressed in olfactory receptor neurons, making them less likely to be infected with SARS-CoV-2 (Butowt et al., 2021; Khan et al., 2021). Furthermore, although SARS-CoV-2 RNA was detected in the olfactory bulb in postmortem COVID-19 brain tissues (Lopez et al., 2022; Serrano et al., 2022), the samples always included the nervus terminalis neurons that express ACE2 (Bilinska et al., 2021; Butowt and von Bartheld, 2022). Without removal of the nervus terminalis from the olfactory bulb, it cannot be differentiated whether the olfactory bulb or the nervus terminalis is infected by the virus based on the RNA data. Furthermore, infection of the olfactory sensory neurons, and the parenchyma of the olfactory bulb by live SARS-CoV-2 was not supported by other evidence (Khan et al., 2021). Reduced volume of the olfactory bulb and tract in COVID-19 patients could be explained by infection of the olfactory epithelium, eliminating crucial support functions performed by the sustentacular cells and the Bowman gland cells, and causing inflammatory or immune reactions in the olfactory epithelium to the olfactory bulb (Liang and Wang, 2021). The death of the infected support cells in the olfactory epithelium is likely to be the cause of OD in COVID-19 instead of the neuroinvasion of the olfactory bulb (Butowt et al., 2023). Taken together, the idea that the olfactory bulb is an important site of neuroinvasion caused by SARS-CoV-2 is unlikely and, at best, highly controversial.

**FIGURE 1:**
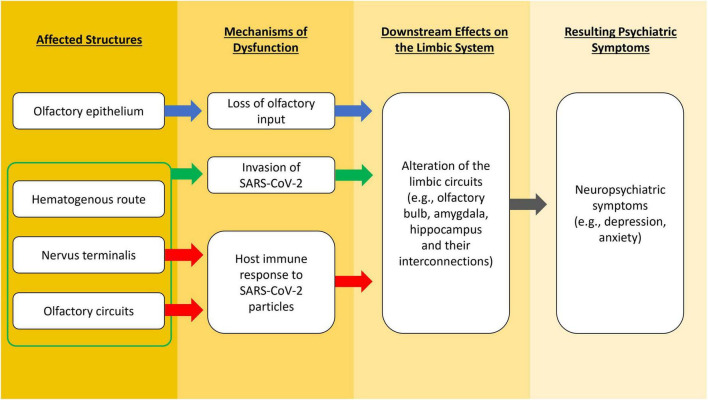
A flowchart illustrating different possible mechanisms that lead to altered functions of the limbic system and the associated neuropsychiatric symptoms in COVID-19.

Alternatively, the nervus terminalis may be considered another path for SARS-CoV-2-induced OD and neural damage *via trans*-synaptic transmission mechanism (Gandhi et al., 2020). The nervus terminalis (or terminal nerve) is closely positioned next to the olfactory nerve, which are located on the anterior and ventromedial surface of the olfactory bulb (Sonne et al., 2017), and this is where the evidence for the presence of SARS-CoV-2 RNA is found (Khan et al., 2021; de Melo et al., 2022). The nervus terminalis neurons express ACE2, which allows the binding of the spike protein from SARS-CoV-2 (Bilinska et al., 2021; Butowt and von Bartheld, 2022). Furthermore, the nervus terminalis projects fibers to the nasal mucosa as well as to the limbic network in the brain, which may provide a direct path for the virus from the neuroepithelium to the CNS (Wirsig-Wiechmann and Lepri, 1991). Abnormalities in limbic areas (e.g., amygdala and entorhinal cortex) are related to depression and anxiety (Charney and Deutch, 1996). Based on these facts, we suggest a novel possibility of CNS manifestation of COVID-19 through the primary attack on the nervus terminalis. Nevertheless, contradictory results from RNA analysis of cerebrospinal fluid in living COVID-19 patients with neuropsychiatric manifestations were reported in another study (Spudich and Nath, 2022), which challenges the neurotropism hypothesis of SARS-CoV-2. Furthermore, neuropathological and autopsy studies show conflicting results in neurotropism of SARS-CoV-2. Some research teams detected viral RNA in the olfactory mucosa, olfactory bulb, olfactory tubercle and other brain regions of COVID-19 patients, and viral proteins in cranial nerves and brainstem (Matschke et al., 2020; Meinhardt et al., 2021), which is observed simultaneously with hyperinflammation in the olfactory bulb and other regions like brainstem. Other teams, however, could not detect the presence of viral RNA or proteins in postmortem brain samples nor specific brain changes related to the virus (Solomon et al., 2020; Fullard et al., 2021). Though neurological manifestations are common in COVID-19 patients, it remains a debate whether SARS-CoV-2 damages the CNS *via* neurotropism, systemic inflammation (Emmi et al., 2023), or both. The abovementioned possible mechanisms that lead to altered functions of the limbic system and the associated neuropsychiatric symptoms in COVID-19 are illustrated in Figure 1.

## 7. Conclusion

In this review, we discussed the possible mechanisms of how SARS-CoV-2 may cause neuropsychiatric symptoms. We speculate SARS-CoV-2 or its particles could attack the nervus terminalis rather than the olfactory pathways and invade other brain regions connected to it through *trans*-synaptic transmission mechanism, which may be a potential cause for the neuropsychiatric symptoms of COVID-19. As an alternative, viral particles may elicit host immune responses, or lack of olfactory input may alter limbic circuits connected to the olfactory system, thereby altering limbic structures which manifest in neuropsychiatric symptoms. COVID-19 is regarded as a multi-systemic disease which may also cause CNS disruption *via* cytokine storm, hyperinflammation, vascular dysfunction and abnormal blood physiology. Ischemic injury remains a major cause of cortical damage and olfactory dysfunction based on our current understanding. Future research is required to elucidate the precise mechanisms by which SARS-CoV-2 causes dysfunction in limbic circuits that manifest as neuropsychiatric symptoms.

## Author contributions

AT, M-KL, BL, SN, and WL contributed to the writing of the manuscript. All authors contributed to the article and approved the submitted version.
